# Decompression alone or fusion in single-level lumbar spinal stenosis with spondylolisthesis? A systematic review and meta analysis

**DOI:** 10.1186/s12891-024-07641-5

**Published:** 2024-09-10

**Authors:** Haiyang Cheng, Gan Luo, Dan Xu, Yuqiao Li, Houzhi Yang, Sheng Cao, Tianwei Sun

**Affiliations:** 1 Tianjin Medical University, Tianjin, 300070 China; 2 Department of Spinal Surgery, Tianjin Union Medical Center, Tianjin, 300121 China; 3 Department of Orthopedics, Chengdu Integrated Traditional Chinese Medicine &Western Medicine Hospital, Chengdu First People’s Hospital, Chengdu, 610016 China; 4 Peking University People’s Hospital, Beijing, 100871 China

**Keywords:** Spinal stenosis, Spondylolisthesis, Decompression, Fusion, Meta analysis

## Abstract

**Purpose:**

The objective of this systematic review and metaanalysis is to compare the efficacy and safety of decompression alone versus decompression plus fusion in single-level lumbar spinal stenosis with spondylolisthesis.

**Methods:**

A comprehensive search of the PubMed, Embase, Cochrane Library, and Ovid Medline databases was conducted to find randomized control trials (RCTs) or cohort studies that compared decompression alone and decompression plus fusion in single-level lumbar spinal stenosis with spondylolisthesis. Operation time; reoperation; postoperative complications; postoperative Oswestry disability index(ODI) scores and scores related to back and leg pain were collected from eligible studies for meta-analysis.

**Results:**

We included 3 randomized controlled trials and 9 cohort studies with 6182 patients. The decompression alone group showed less operative time(*P* < 0.001) and intraoperative blood loss(*p* = 0.000), and no significant difference in postoperative complications was observed in randomized controlled trials(*p* = 0.428) or cohort studies(*p* = 0.731). There was no significant difference between the other two groups in reoperation(*P* = 0.071), postoperative ODI scores and scores related to back and leg pain.

**Conclusions:**

In this study, we found that the decompression alone group performed better in terms of operation time and intraoperative blood loss, and there was no significant difference between the two surgical methods in rate of reoperation and postoperative complications, ODI, low back pain and leg pain. Therefore, we come to the conclusion that decompression alone is not inferior to decompression and fusion in patients with single-level lumbar spinal stenosis with spondylolisthesis.

**Supplementary Information:**

The online version contains supplementary material available at 10.1186/s12891-024-07641-5.

## Background

Lumbar spinal stenosis refers to a series of clinical symptoms caused by nerve root compression in the lumbar spinal canal due to various reasons [1, 2]. Lumbar spondylolisthesis refers to the mutual displacement between adjacent vertebral bodies, generally defined as the anterior displacement of the upper vertebral body relative to the lower vertebral body [3]. Lumbar spinal stenosis with spondylolisthesis is a common disease in spine surgery. The main clinical symptoms of this disease are low back pain, hip pain, lower limb pain and numbness, and neurogenic claudication, which affects the daily life of patients [4]. Non-surgical treatment including physical therapy, drug therapy or acupuncture can help patients relieve symptoms to a certain extent, but when the condition is serious and conservative treatment is ineffective, doctors will consider surgical treatment for patients [5, 6]. Previous studies have confirmed that surgical treatment has a better prognosis and significantly improved quality of life compared with conservative treatment [7, 8]. With the development of implants, interbody fusion through a variety of approaches has gradually been favored by clinicians. In some regions, spinal canal decompression combined with instrument fusion has accounted for more than 90% of the treatment of lumbar spondylolisthesis [9, 10]. However, with the increasing use of decompression and fusion surgery in the case of lumbar spinal stenosis with spondylolisthesis, more and more problems have begun to appear, such as prolonged operation and hospitalization time, increased treatment costs, increased postoperative complications and reoperation rate, and other adverse consequences [11–13]. Therefore, the surgical treatment of lumbar spinal stenosis with spondylolisthesis is controversial [6]. The most controversial were three randomized controlled trials on the selection of surgical procedure for single-level lumbar spinal stenosis with spondylolisthesis. Inose concluded that f decompression plus fusion was not superior to decompression alone [14], and the RCT conducted by Austevoll reported that decompression alone was not inferior to decompression plus fusion [15]. However, Ghogawala suggested that decompression plus fusion should be chosen over decompression alone in the surgical treatment of single-level lumbar spinal stenosis with spondylolisthesis [16]. Two systematic reviews [17, 18] did not recommend decompression plus fusion in patients with lumbar spinal stenosis and spondylolisthesis. However, there is no systematic review or meta analysis on the advantages and disadvantages of decompression alone or decompression plus fusion in the surgical treatment of single-level lumbar spinal stenosis with spondylolisthesis. Therefore, this article mainly aimed at the patients with single-level lumbar spinal stenosis with spondylolisthesis, whether fusion surgery is necessary after decompression surgery.

## Materials and methods

This review protocol was registered with PROSPERO(CRD42023399298) and followed the Preferred Reporting Items of Systematic Reviews and Meta-analysis guidelines [19].

### Search strategy

Two investigators independently searched the following databases (inception to Dec 2022): PubMed, Embase, Cochrane Library, Web of Science, and Ovid Medline databases. The search strategy follows PICOS principles: (1) Participants: Patients with single-level lumbar spinal stenosis with spondylolisthesis. (2) Intervention(D group): All decompression procedures for spinal stenosis, including minimally invasive or open laminectomy, unilateral laminectomy and bilateral decompression, etc. (3) Comparison(F group): Additional arthrodesis is performed on the basis of the above decompression procedures, including Interbody fusion or/and posterolateral fusion et al. (4) Outcomes: The study should include at least one of the following data: Operation time, intraoperative blood loss, reoperation, complications, Oswestry disability index(ODI) socres, and scores related to back or leg pain (5) Study design: Observational studies and randomized control trials were eligible.

The electronic search strategy used the following keywords: “spinal stenosis”, spondylolisthesis”, “decompression”, “fusion”. The search terms were adjusted according to the characteristics of each database, and we also examined the reference lists of the screened full-text studies to identify additional trials that might be eligible. And a third reviewer was consulted when the two reviewers could not reach a consensus.

### Eligibility criteria

Inclusion criteria were randomized clinical trials and cohort studies written in English that compared the decompression alone versus decompression plus fusion in patients with single-level lumbar spinal stenosis with spondylolisthesis. We excluded reports surgery with more than one level, case reports, case series, commentaries, practice guidelines, systematic reviews and meta-analysis. In addition, duplicate studies with the same cohort or studies considered by consensus to be of low quality were excluded.

### Data collection

Data were extracted from the included studies as follows: (1)study design: first author, publication region, publication time, and study type; (2) sample demographics: number of patients, age, sex, and disease diagnosis; (3) surgery details: methods of decompression alone and decompression plus fusion, operation time, intraoperative blood loss; (4) analysis variables: reoperation and postoperative complications, postoperative Oswestry disability index(ODI) scores and scores related to back or leg pain. In case of absence of data, variances were transformed or estimated using the recommendations in Sect. 6.5.2 in the Cochrane Handbook for Systematic Reviews of Interventions [20]. We resolved disagreements for data extraction through discussion, or with arbitration by a third reviewer if necessary.

### Assessment of risk of bias

The Cochrane Collaboration’s tool was used by two reviewers to independently evaluate the included RCTs for potential bias. And the bias risk of cohort studies was evaluated by the Newcastle-Ottawa scales, The quality of cohort studies was evaluated according to the Newcastle-Ottawa scale, with scores above 7 (including 7) of high quality.

### Statistical analysis

The continuous variables were estimated by weighted mean difference (WMD), and for dichotomous variables, odds ratios (ORs) with a 95% confdence interval (CI) were calculated. The statistical heterogeneity of the pooled results was determined using the I² statistic. For this meta-analysis, we used the fixed-effect model when I² was greater than 50%, and if I² was less than 50%, a random-effect model was applied. The meta-analysis results were considered statistically significant when the *p* value < 0.05. And the magnitude of publication bias was estimated by Begg’ and Egger’ test. The meta-analysis was performed using STATA 16.0 (StataCorp, College Station, TX, USA).

## Results

### Search results

**Fig. 1:**
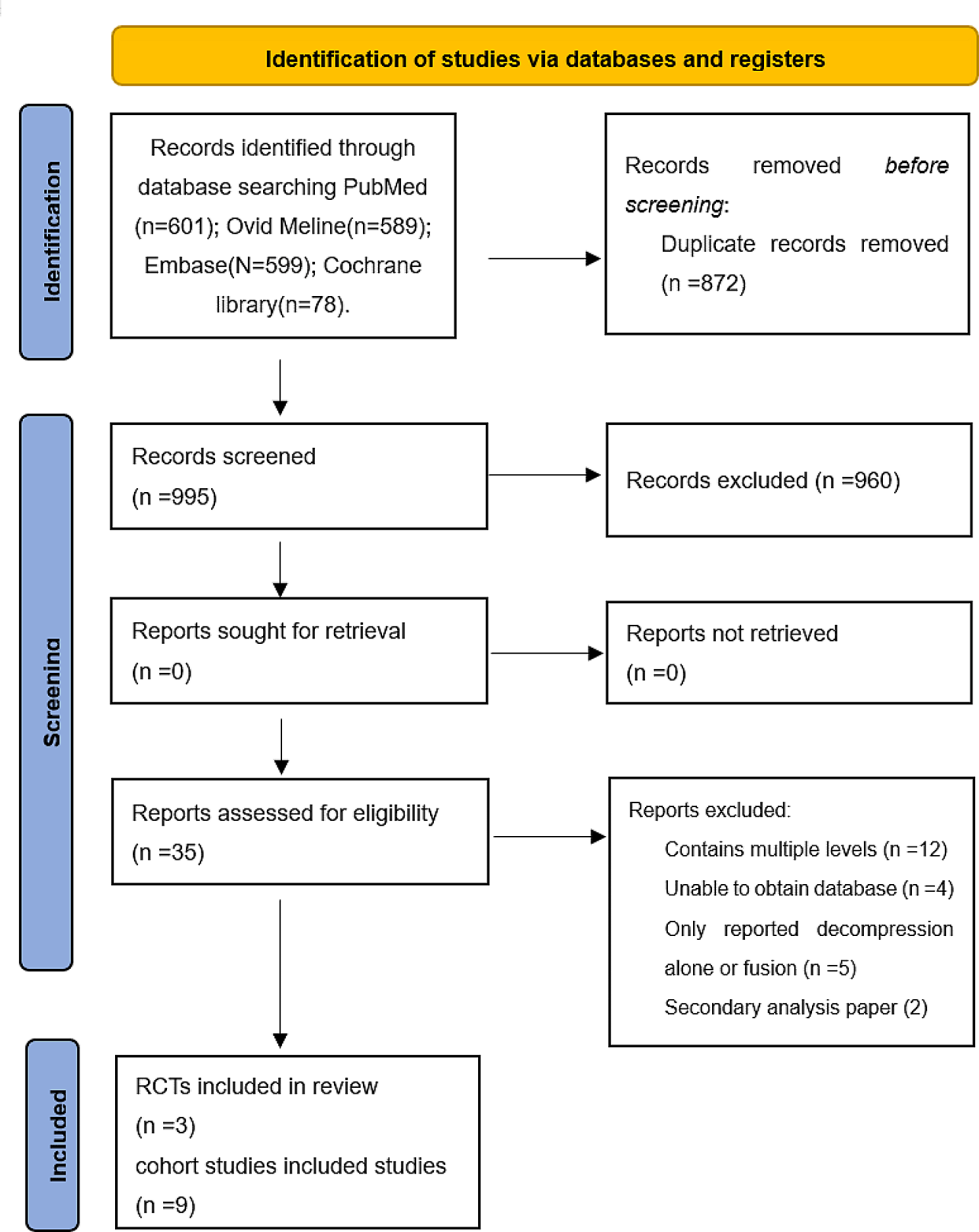
Flow diagram for the selection of studies (PRISMA)

A total of 131 articles from PubMed, Embase, Cochrane Library, and Ovid Medline databases were initially identified. The exact number of articles identified in each database is as follows: PubMed (*n* = 601), Embase (*n* = 599), the Cochrane library (*n* = 78), Ovid Meline(*n* = 589). 872 articles were excluded because of duplication, and 960 studies were excluded by screening the titles and abstracts for: irrelevant studies, case reports, non-comparative studies and review articles. Leaving 35 articles that underwent a comprehensive full-text analysis. Finally, 3 randomized controlled trials [15, 16, 21] and 9 cohort studies [22–29] were included studies in the final meta-analysis. The flow chart used for the new systematic review according to PRISMA 2020 is shown in Fig. 1.

### Study characteristics and quality assessment

A total of 6182 patients were enrolled in the 3 randomized controlled trials and 8 cohort studies. The decompression alone group(D group) included 2339 participants, and the decompression plus fusion group(F group) included 3783 patients. The characteristics of the included studies are presented in Table 1. A summary of the risk of bias assessment of the RCTs is displayed in Fig.S1 and the risks of bias of the included cohort studies are displayed in Table S1.

**Table 1:** The characteristics of the included 11 studies

Author(Year)	Country	Study Design		Participants number	Age(SD)	Surgical methods	outcome
Austevoll(2021)	Norway	multicentric RCT	D	133	66(7.4)	posterior decompression that preserved the midline structures	operation time/ Intraoperative Blood loss/Reoperation/ Postoperative Complications/ODI scores/ scores related to back and leg pain(NRS)
			F	129	66.5(7.9)	posterior decompression followed by implantation of pedicle screws with rods and bone grafting	
Badhiwala(2021)	Canada	multicentric cohort studies	D	802	64.4(11.6)	Laminectomy	operation time/ Reoperation/ Postoperative Complications
			F	1002	62.7(12.1)	laminectomy plus posterolateral fusion	
Ghogawala(2016)	USA	multicenter RCT	D	35	66.5(8.0)	complete laminectomy with partial removal of the medial facet joint	operation time/ Intraoperative Blood loss/ Reoperation/ Postoperative Complications/ ODI scores
			F	31	66.7(7.2)	laminectomy with pedicle screws and titanium alloy rods, with a iliac bone graft	
Hua(2021)	China	unicentric cohort studies	D	24	59(7.9)	lumbar endoscopic unilateral laminotomy bilateral decompression	operation time/ Intraoperative Blood loss/ Reoperation/Postoperative Complications/ ODI scores/ scores related to back and leg pain(VAS)
			F	36	59.9(8.6)	minimally invasive transforaminal lumbar interbody fusion	
Inose(2018)	Japan	multicentric RCT	D	29	63.4(8.7)	wide fenestration and preserve the midline structure	operation time/ Intraoperative Blood loss/ Reoperation
			F	31	63.5(6.8)	decompression and posterolateral fusion followed by implantation of pedicle screws with rods and bone grafting	
Joelson(2022)	Sweden	multicentric cohort studies	D	144	69.4(9.4)	decompression	Reoperation/ Postoperative Complications/ ODI scores /scores related to back and leg pain(NRS)
			F	228	63.9(8.9)	Posterolateral fusion/ Posterior Lumbar Interbody Fusion/ Transforaminal Lumbar Interbody Fusion	
Joelson(2021)	Sweden	multicentric cohort studies	D	597	69(9.9)	decompression	Reoperation
			F	1338	65(9.1)	Posterolateral fusion/ Posterior Lumbar Interbody Fusion/ Transforaminal Lumbar Interbody Fusion	
Kimura(2019)	Japan	multicentric cohort studies	D	28	70(12.8)	microendoscopic muscle-preserving interlaminar decompression	operation time/ Reoperation/ Postoperative Complications
			F	50	68.5(9.3)	Posterior Lumbar Interbody Fusion	
Matsudaira(2005)	Japan	unicentric cohort studies	D	18	68.0(7.0)	laminoplasty with preserving the integrity of the midline structure	Reoperation/ Postoperative Complications
			F	19	67.0(7.0)	laminectomy with posterolateral fusion and pedicle screw instrumentation	
Sigmundsson(2015)	Sweden	multicentric cohort studies	D	245	73.5(9.94)	open decompression	ODI scores / scores related to back and leg pain(VAS)
			F	594	70.0(8.92)	Posterolateral fusion	
Yagi(2018)	Japan	multicentric cohort studies	D	59	68.5(9.3)	laminotomy	Reoperation/ ODI scores
			F	40	66.7(7.1)	Posterior Lumbar Interbody Fusion/Transforaminal Lumbar Interbody Fusion	
Austevoll(2020)	Norway	multicentric cohort studies	D	285	64.6(9.8)	Microdecompression	operation time/ Postoperative Complications/ODI scores/ scores related to back and leg pain(NRS)
			F	285	64.8(9.2)	Decompression plus instrumented fusion	

*D*:decompression group; *F*: decompression plus fusion group; *ODI*: Oswestry disability index; *VAS*: visual analogue scale; *NRS*: Numeric Rating Scale

### Meta-analysis results

#### Operation time

**Fig. 2:**
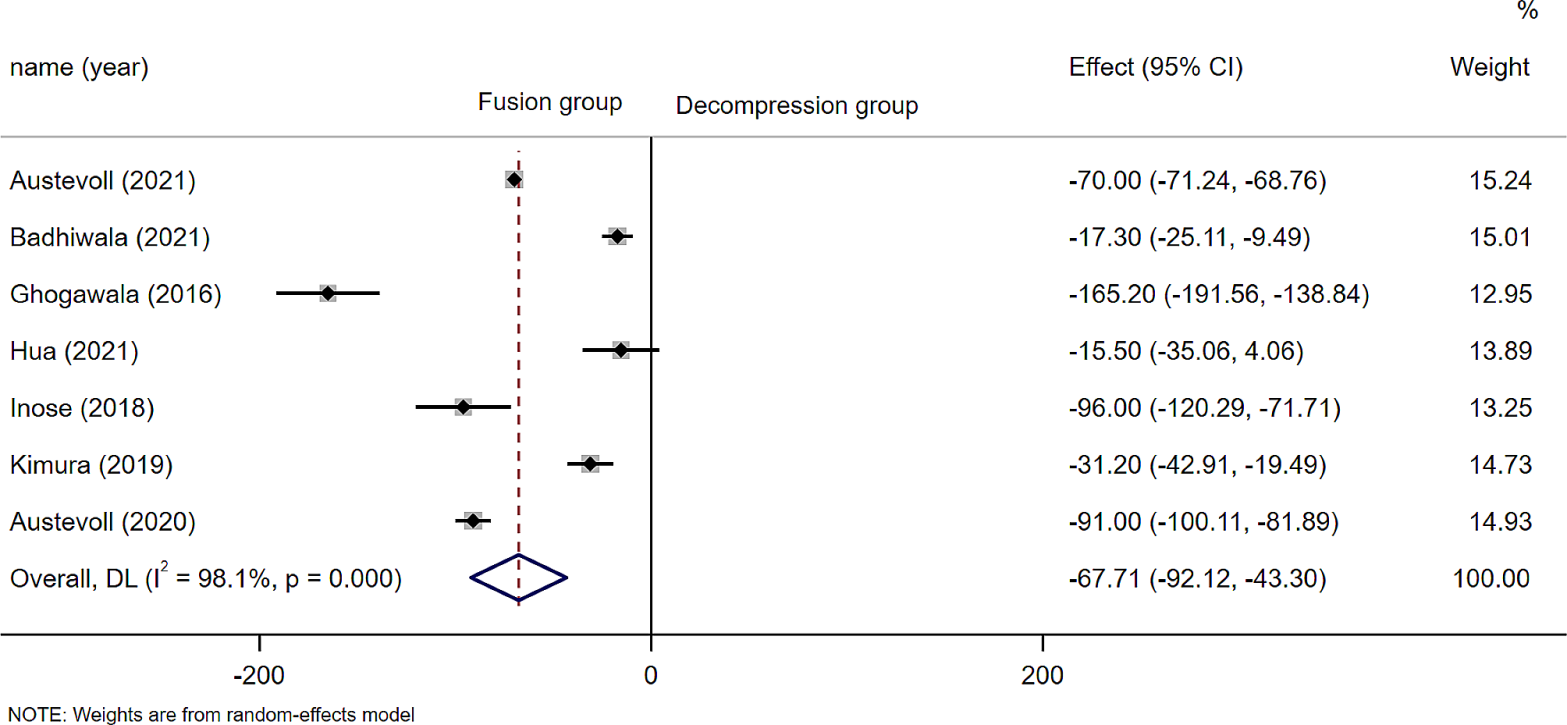
Forest plot of operation time; CI: Confidence Internal

Seven studies (*n* = 2936 patients; 1344 in the D group and 1592 in the F group) provided operation time, and there was statistically significant difference between the two groups regarding Operation time(WMD − 67.71; 95% CI − 92.12 to − 43.30, *P* = 0.000; I²=98.1%, *p* = 0.000)(Fig. 2). The heterogeneity was not reduced after subgroup analysis and and included studies (Except chen reported that the same operation time in both groups) reported that the operation time in the decompression alone group was shorter than that in the decompression plus fusion group.

### Intraoperative blood loss

**Fig. 3:**
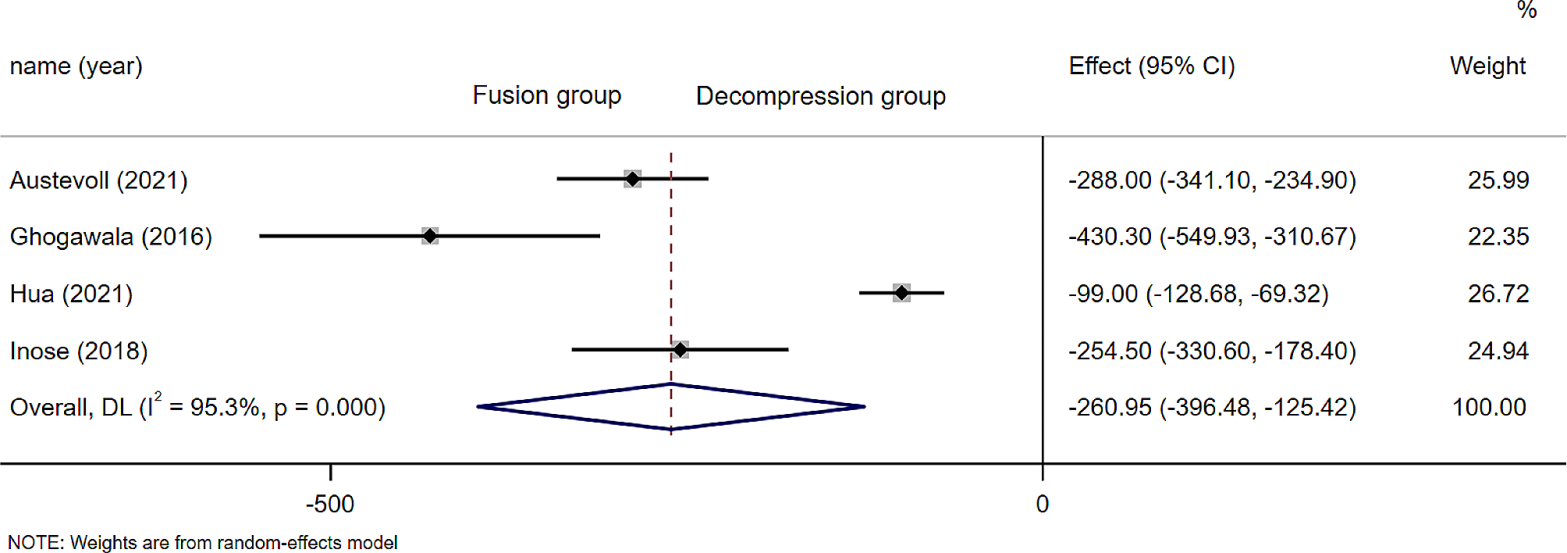
Forest plot of intraoperative blood loss

A total of 4 studies reported the intraoperative blood loss (*n* = 447 patients; 220 in the D group and 247 in the F group). Statistically significant difference was observed in D group and F group (WMD − 260.95; 95% CI − 396.48 to − 125.42, *P* = 0.000; I² =95.3%, *p* = 0.000)(Fig. 3). Since Kimura(2019) [26] did not provide standard deviation, this part of the combined effect value was not calculated. However, the intraoperative blood loss reported by Kimura(2019) was also less in the decompression alone group than in the decompression plus fusion group. Subgroup analysis was based on surgical procedures (open or minimally invasive), and the heterogeneity decreased after excluding Hua (2021) [23](WMD − 310.04; 95% CI − 389.53 to − 230.54 *P* = 0.000; I² =67%, *p* = 0.048)(Fig. S2).

### Reoperation

**Fig. 4:**
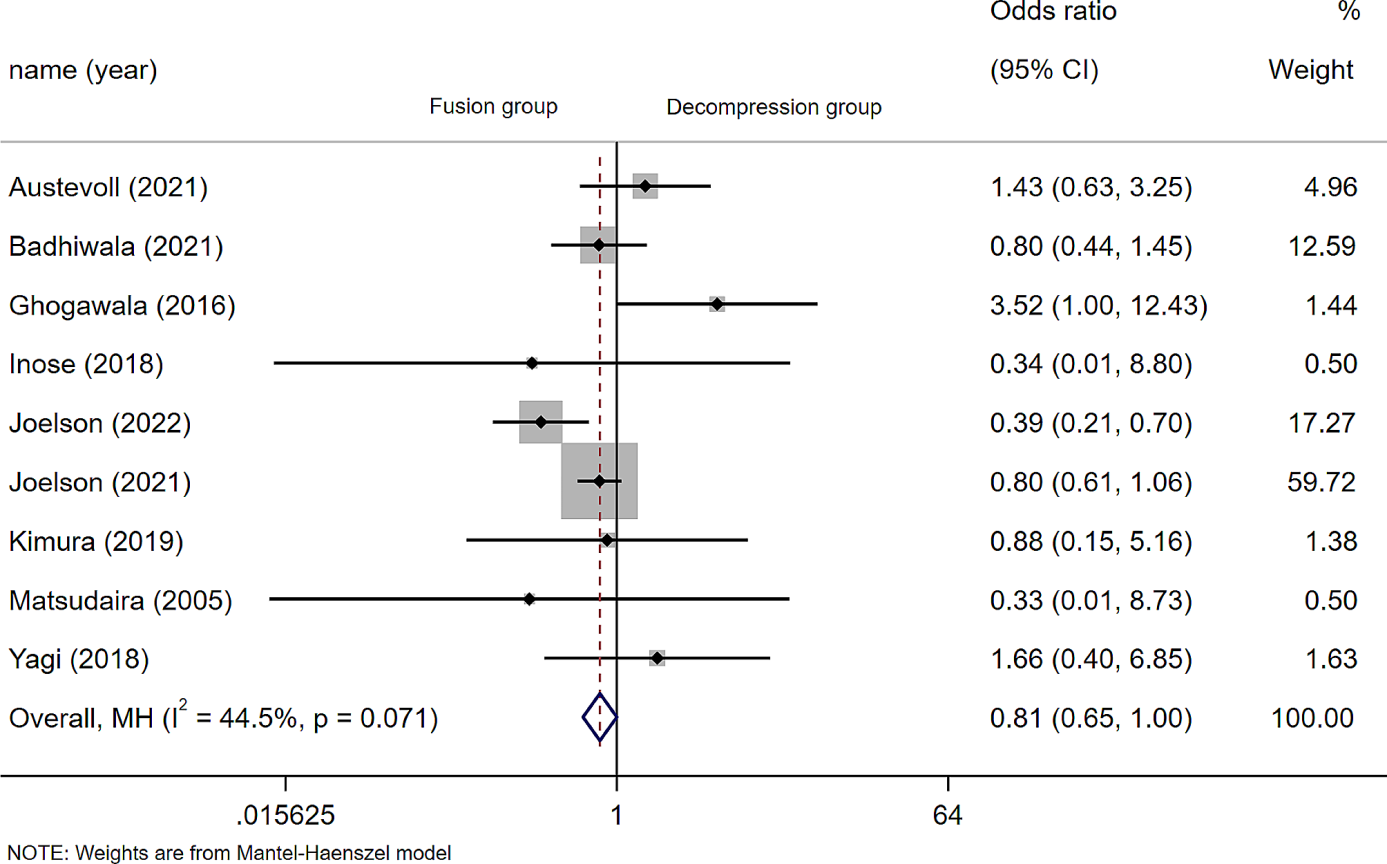
Forest plot of reoperation

A total of 10 articles reported the occurrence of reoperation after surgery, but no reoperation was observed in the two groups by hua2021 during the two-year follow-up period, so a total of 9 articles (*n* = 4773 patients; 1953 in the D group and 2820 in the F group) were used for Meta-analysis. There was no statistically significant difference in reoperation between the D group and F group (OR 0.87; 95% CI 0.58 to 1.30, *P* = 0.493; I² =44.5%, *p* = 0.071) (Fig. 4), Subgroup analysis by article type (cohort study or RCT) and duration of follow-up (2 years or more) reduced heterogeneity, but no between-group differences in reoperation were observed (Fig. S3/Fig. S4).

### Postoperative complications

**Fig. 5:**
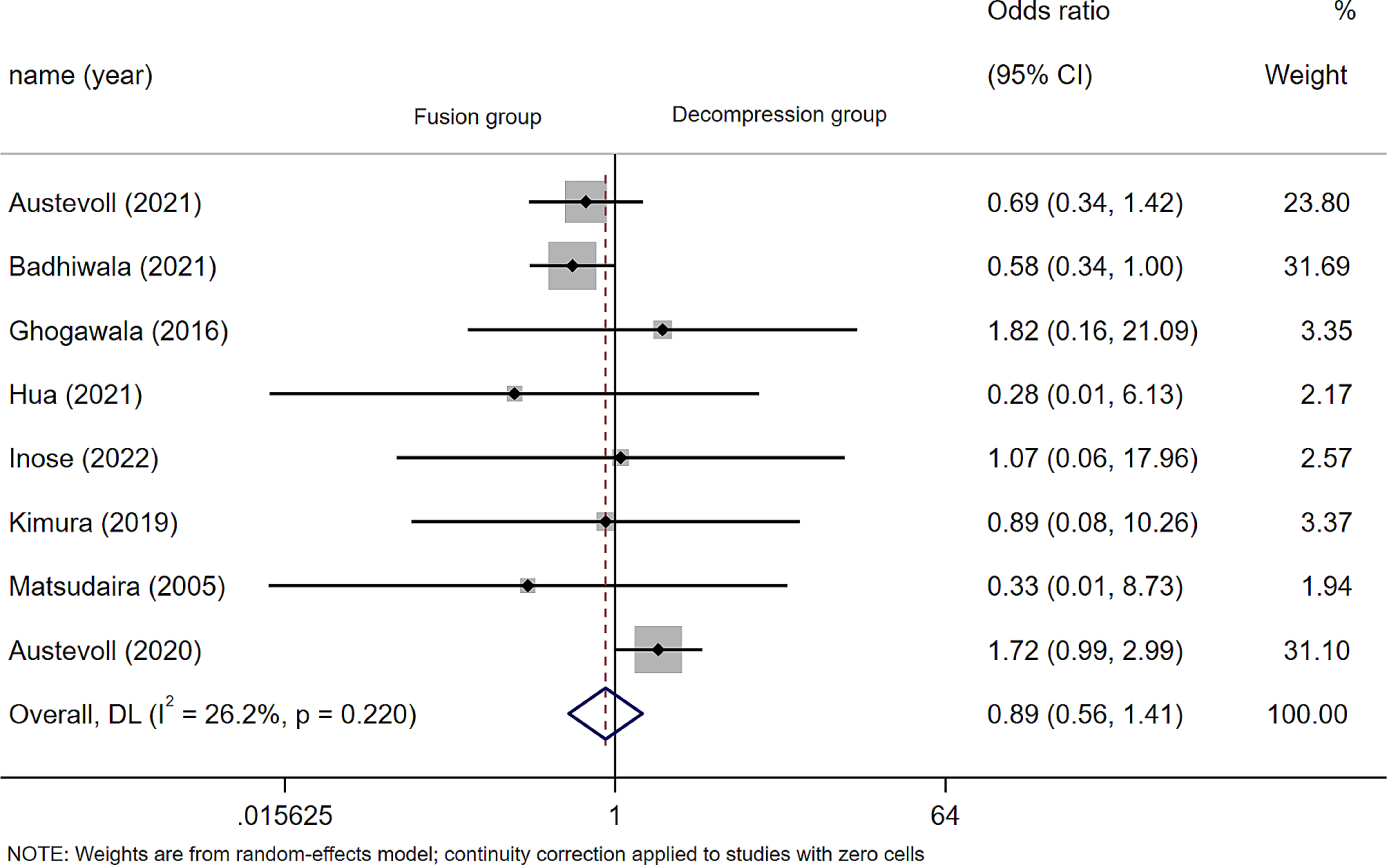
Forest plot of postoperative complications

Eight articles(*n* = 2746 patients; 1275 in the D group and 1741 in the F group) reported postoperative complications, which did not include changes in neurological function, only included infection, hematoma, or other systemic complications such as pneumonia, urine retention, etc. The results showed that the incidence of postoperative complications in D group was lower than that in F group (OR 0.89; 95% CI 0.56 to 1.41, *P* = 0.612; I² = 26.2%, *p* = 0.220) (Fig. 5). Subgroup analysis by article type showed no significant statistical differences between the two groups in the cohort studies (OR 0.89; 95% CI 0.56 to 1.41, *P* = 0.731; I² = 53.2%, *p* = 0.074), or the randomized controlled trials(OR 0.77; 95% CI 0.39 to 1.49, *P* = 0.433; I² = 0.0%, *p* = 0.738)(Fig. S5).

### Oswestry disability index (ODI) scores

**Fig. 6:**
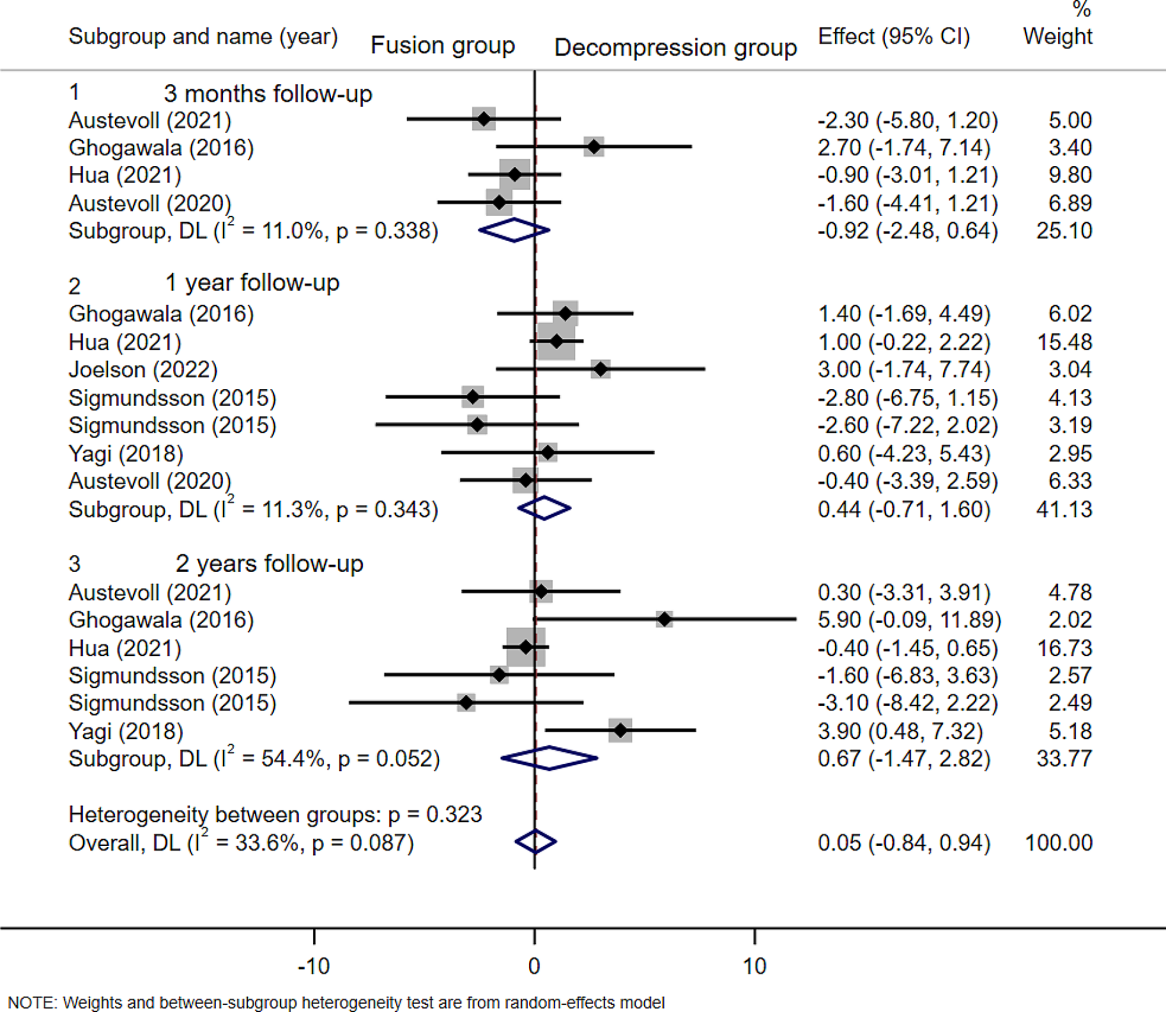
Forest plot of postoperative oswestry disability index (ODI) scores

Seven articles(*n* = 2268 patients; 925 in the D group and 1343 in the F group) provided baseline Oswestry disability index scores. Sigmundsson(2015) [28] grouped participants according to preoperative low back pain and back pain, so to avoid the bias caused by grouping within the study, the articles were divided into two groups(BP < LP for Sigmundsson1 and BP > LP for Sigmundsson2) for analysis. There was no statistically significant difference in ODI scores between decompression alone group and decompression plus fusion group at the follow-up time of 3 months(WMD − 0.92; 95% CI -2.48 to 0.64, *p* = 0.246; I² =11%, *p* = 0.338), 1 year(WMD 0.44; 95% CI -0.71 to 1.60, *p* = 0.453; I² =11.3%, *p* = 0.343), and 2 years(WMD 0.67; 95% CI -1.14 to -0.69, *p* = 0.908; I² =54.4%, *p* = 0.052)(Fig. 6).

### Back pain and leg pain

**Fig. 7:**
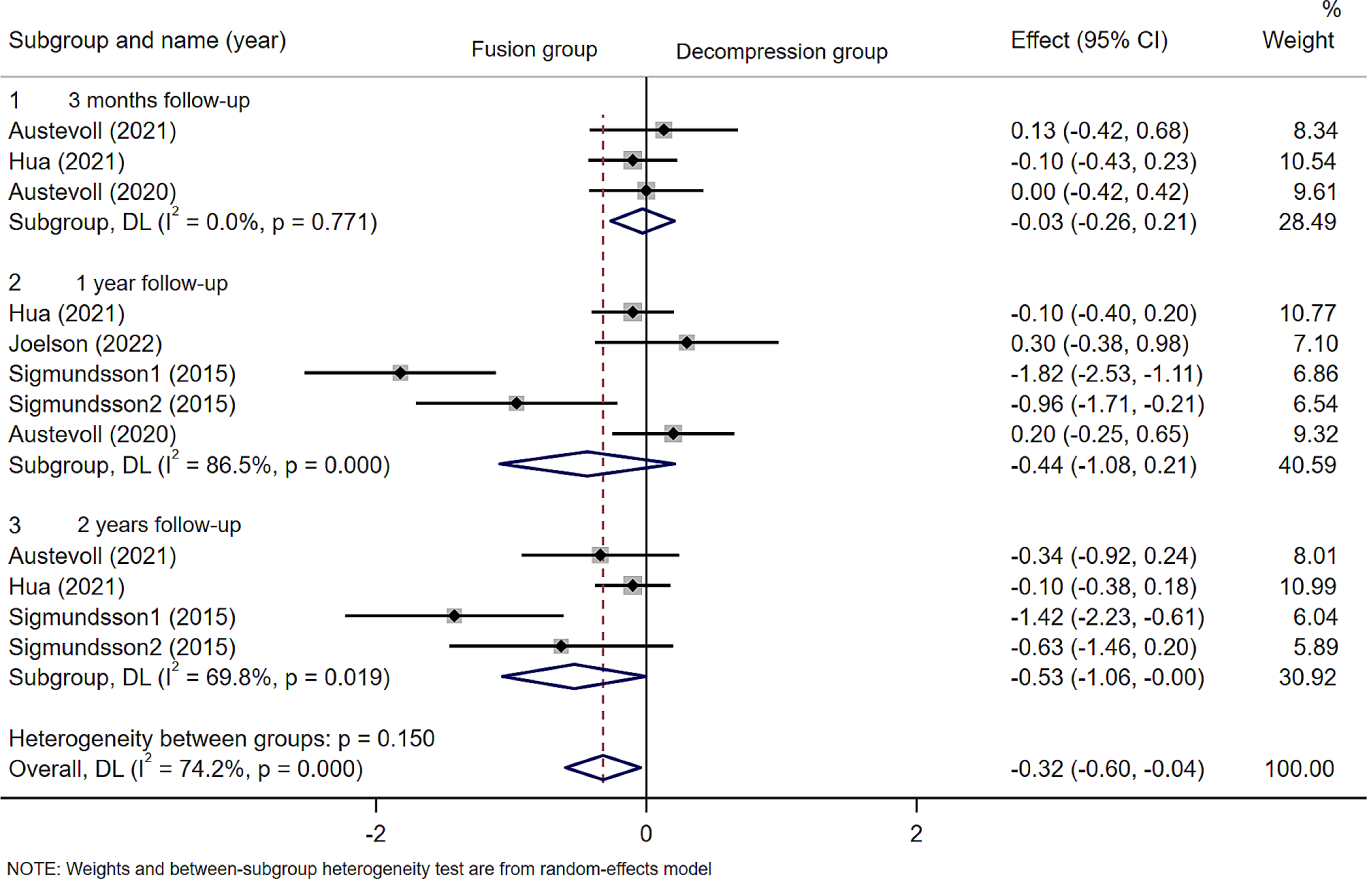
Forest plot of scores related to back pain

**Fig. 8:**
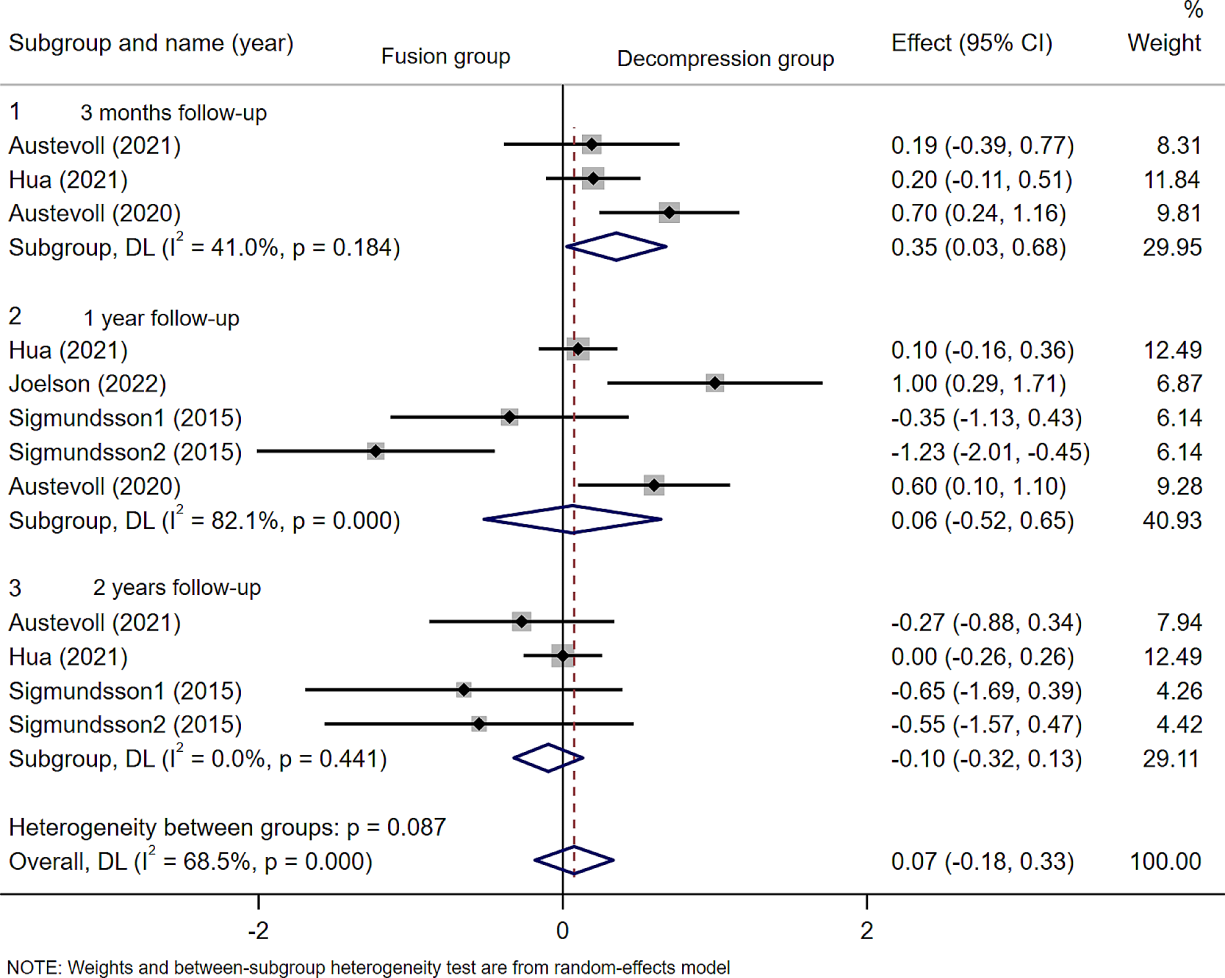
Forest plot of scores related to leg pain

A total of five included articles(*n* = 2103 patients; 915 in the D group and 1188 in the F group) reported scores related to back pain and leg pain, including Numeric Rating Scale(NRS) and visual analogue scale(VAS)scores. As with ODI scores, Sigmundsson2015 was divided into two groups for analysis. The results of the meta-analysis showed that there were no statistically significant differences in back pian at 3 months(WMD − 0.03; 95% CI -0.26 to 0.21, *p* = 0.821; I² =0.0%, *p* = 0.771), 1 year(WMD − 0.44; 95% CI -1.08 to 0.21, *p* = 0.186; I² =86.5%, *p* = 0.000) and 2 years(WMD − 0.53; 95% CI -1.06 to 0.00, *p* = 0.05; I² =69.8%, *p* = 0.019)( Fig. 7) after surgery. And for leg pain, no statistically significant differences were observed at 3 months (WMD 0.35; 95% CI -0.03 to 0.68, *p* = 0.034; I² =41%, *p* = 0.184), 1 year (WMD − 0.06; 95% CI -0.52 to 0.65, *p* = 0.831; I² =82.1%, *p* = 0.000) and 2 years (WMD − 0.10; 95% CI -0.32 to 0.13, *p* = 0.410; I² =0.0%, *p* = 0.441) postoperatively (Fig. 8).

### Publication bias

The potential publication bias was evaluated by Begg’ and Egger’ test, and the results showed that there was no obvious publication bias in all indicators. The results are shown in the table S2.

## Discussion

A total of 6182 patients were included in this study, including 2339 patients in the decompression alone group and 3783 patients in the decompression plus fusion group. The operation time, intraoperative blood loss, postoperative complications, Oswestry disability index (ODI) scores and scores related to back and leg pain were analyzed. We found that decompression alone was superior to decompression plus fusion with respect to operative time and intraoperative blood loss, and there were no other differences between the two groups.

In terms of operation time and intraoperative blood loss, the conclusions were consistent with the results of all the included studies with relevant indexes: the operation time and intraoperative blood loss of the decompression group alone were less, and the reason for the higher heterogeneity was considered to be related to the different decompression and fusion methods, as well as the understanding of the standard operation procedures and the surgeons in various regions. In addition to decompression, the surgeon needs to perform cage or screw implantation, as well as intraoperative X-ray projection and other operations, which will prolong the operation time and intraoperative bleeding, which is consistent with various studies and systematic reviews [11, 17, 18].

No significant heterogeneity was observed in the reoperation either in the overall effect size or in the subgroup analysis. There were no significant differences in reoperation rates and postoperative complications between the decompression alone group and the decompression plus fusion group. Sato [30] found that among patients with lumbar spondylolisthesis, decompression alone was awssociated with more reoperation rates after at least 5 years of follow-up. But other studies [31, 32] found no gap between the two groups in degenerative spondylolisthesis patients. Wei [33] showed in a meta-analysis that no significant difference was found in reoperation between the two groups in short-term (< 4 years) or long-term (> 4 years). We did not find a difference in the randomized controlled trials or in the cohort studies, which is consistent with our finding that there was no difference between two groups in postoperative complications [34]. In addition to further progression of spondylolisthesis, facet joint invasion and degeneration of adjacent joints are important causes of reoperation [35]. Therefore, to reduce the reoperation rate of instrumental fusion surgery, attention should be paid to the accuracy of intraoperative pedicle screw implantation, and Robot-Assisted pedicle screw implantation is an effective way.

In terms of postoperative scores related to back and leg pain, we found no significant difference between the two groups at 3 months, 1 year, and 2 years after surgery. Similarly, Försth [11] found no significant difference between the two groups at 12 months in patients with degenerative spondylolisthesis patients. Similar findings have been confirmed by multiple studies [31, 36]. However, Chan [37, 38] found in two studies that patients with lumbar spondylolisthesis had better improvement in ODI scores in the fusion group than in the decompression alone group. Among the included studies in this paper, one RCT [36] and four cohort studies [15, 23, 24, 29, 36] showed no significant difference in ODI scores between the two groups. while Sigmundsson’s [28] study found that fusion surgery could bring better improvement in ODI scores, but also proposed that the statistical difference may not reach the minimum clinically significant difference. Similarly, Ghogawala [16] reported that the fusion group showed a higher ODI score improvement, but there was no statistical difference.

Previous studies [31, 38] have found that in patients with lumbar spondylolisthesis, there was no significant difference in back pain or leg pain between the two groups with or without fusion. Although the methods used to evaluate back pain and leg pain were slightly different in this study, the basic principles were the same and were analyzed as in other studies [33]. In patients with lumbar degenerative spondylolisthesis or spinal stenosis, Försth [11] found no difference between the two groups after surgery, while the multi-center cohort study conducted by Austevoll [36] et al. found that for patients with lumbar degenerative spondylolisthesis, the fusion group showed a higher improvement in low back pain at 12 months, and Chan [37] also found that the fusion group was associated with better improvement in back pain at 24 months. However, there was no significant difference in leg pain between the two groups. All the articles included in this study reported no significant difference in scores related to back and leg pain in patients with single-level lumbar spinal stenosis with spondylolisthesis [15, 23, 24, 28].

Since Briggs [39] first described lumbar fusion in 1944, lumbar interbody fusion has been widely used to treat a range of spinal diseases. It can stabilize the vertebral body in cases of instability caused by trauma, infection, degeneration, deformities, or iatrogenic factors [40]. As mentioned above, spinal fusion surgery has become the preferred surgical method for clinical surgeons as an auxiliary means of decompression. Although some scholars have always questioned the necessity of adding spinal fusion surgery after decompression, it has not attracted attention. However, in 2016, Ghogawala and Försth presented different research conclusions in their articles respectively, this necessity became the focus of debate. The relevant guidelines recommend the use of fusion surgery in patients with lumbar spinal stenosis and degenerative spondylolisthesis [41], while it is not recommended for patients with spinal stenosis without spondylolisthesis [42]. However, in this paper, we believe that fusion surgery should not be used routinely in the case of single-level lumbar spinal stenosis with degenerative spondylolisthesis, but research should seek to improve knowledge about individualized surgical treatment. One can not rule out that subgroups of patients can benefit from fusion surgery.

### Limitations of the study

This study has some limitations. First of all, there is great heterogeneity in the evaluation indicators such as operation time and intraoperative loss, which is considered to be due to the differences between the operations and the differences between the operators, so it may be necessary to consider the consistency of surgical procedures in more studies. Secondly, for postoperative Scores related to back and leg pain, the pain assessment methods were different among different studies, including Numeric Rating Scale(NRS) and visual analogue scale(VAS)scores. Although the basic principles were consistent, there were confounding factors when analyzing the combined effect size in the analysis of low back pain and leg pain. Thirdly, most of the articles included in this study were cohort studies, and only three randomized controlled trials were included. The level of evidence was low, and there may be many potential confounding factors affecting the results, so a large number of randomized controlled trials are needed to obtain the results. Fourth, this study only focused on the most common type of lumbar spondylolisthesis, single-level spondylolisthesis with spinal stenosis, without exploring multi-level spondylolisthesis or multi-level spinal stenosis. More complex types of spondylolisthesis should be paid attention to in the later stage.

## Conclusion

The results of this study showed that in the patients with single-level lumbar spinal stenosis and degenerative spondylolisthesis, the operation time and intraoperative blood loss of decompression alone were significantly better than those of decompression plus fusion. In terms of postoperative complications, there was no significant difference between decompression alone group and decompression plus fusion group in general, the subgroup analysis of the article type suggests that the incidence of postoperative complications in the cohort study is lower in the decompression alone group than in the decompression plus fusion group. There was no significant difference in reoperation, postoperative ODI scores and scores related to back and leg pain between decompression alone group and decompression plus fusion group. Therefore, this study concluded that decompression alone was superior to decompression plus fusion with respect to operative time and intraoperative blood loss, and there were no other differences between the two groups in patients with single-level lumbar spinal stenosis and degenerative spondylolisthesis. In addition, considering the longer operation cost and more intraoperative blood loss for decompression plus fusion, we recommend that decompression alone be preferred in patients with single-level lumbar spinal stenosis with degenerative spondylolisthesis.

## Electronic supplementary material

Below is the link to the electronic supplementary material.

Supplementary Material 1

## Data Availability

The data of this study are included in the manuscript and supplementary materials.

## Abbreviations

CI: Confdence interval
NRS: Numeric Rating Scale
ODI: Oswestry disability index
ORs: Odds ratios
PRISMA: Preferred Reporting Items for Systematic reviews and Meta-Analyses
RCTs: Randomized control trials
VAS: Visual analogue scale
WMD: Weighted mean difference
