# Supplementary material for: Decompression alone or fusion in single-level lumbar spinal stenosis with spondylolisthesis? A systematic review and meta analysis
Source: BMC Musculoskelet Disord. 2024 Sep 10;25:726. doi: 10.1186/s12891-024-07641-5 (PMC11386329; doi:10.1186/s12891-024-07641-5)
Supplement: Supplementary file 1 — Supplementary Material 1 [file 12891_2024_7641_MOESM1_ESM.docx]

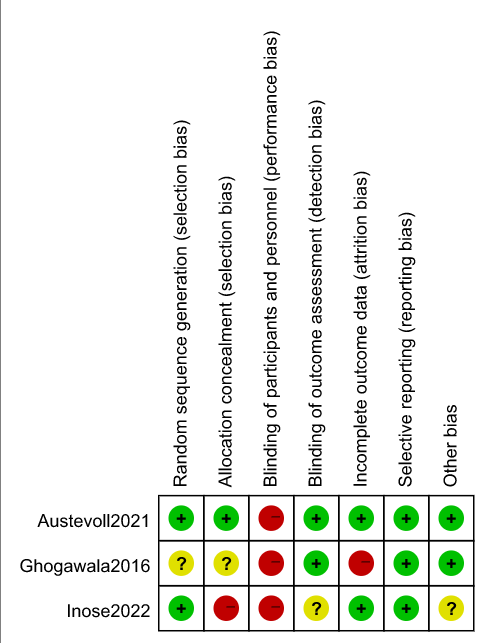


Fig.S1 | Risk of bias of included RCTs


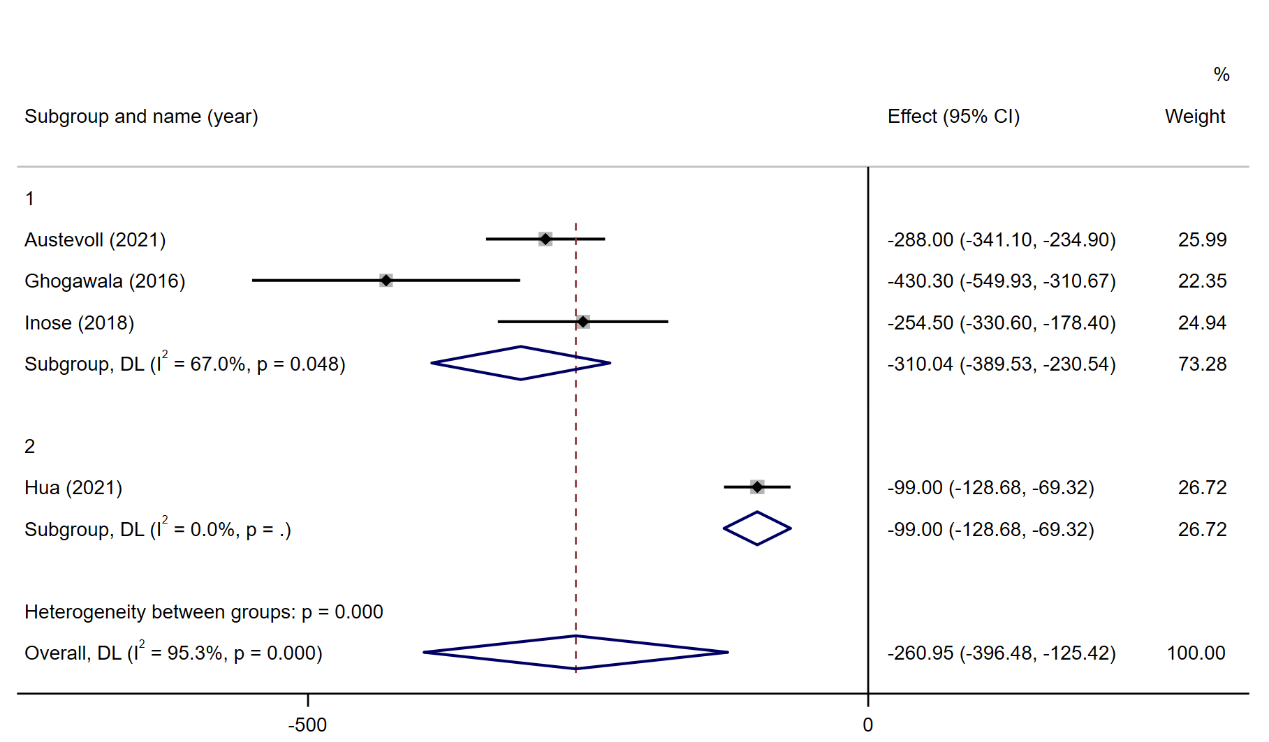


Fig.S2 Subgroup forest plot of intraoperative blood loss based on surgical procedures (open or minimally invasive)


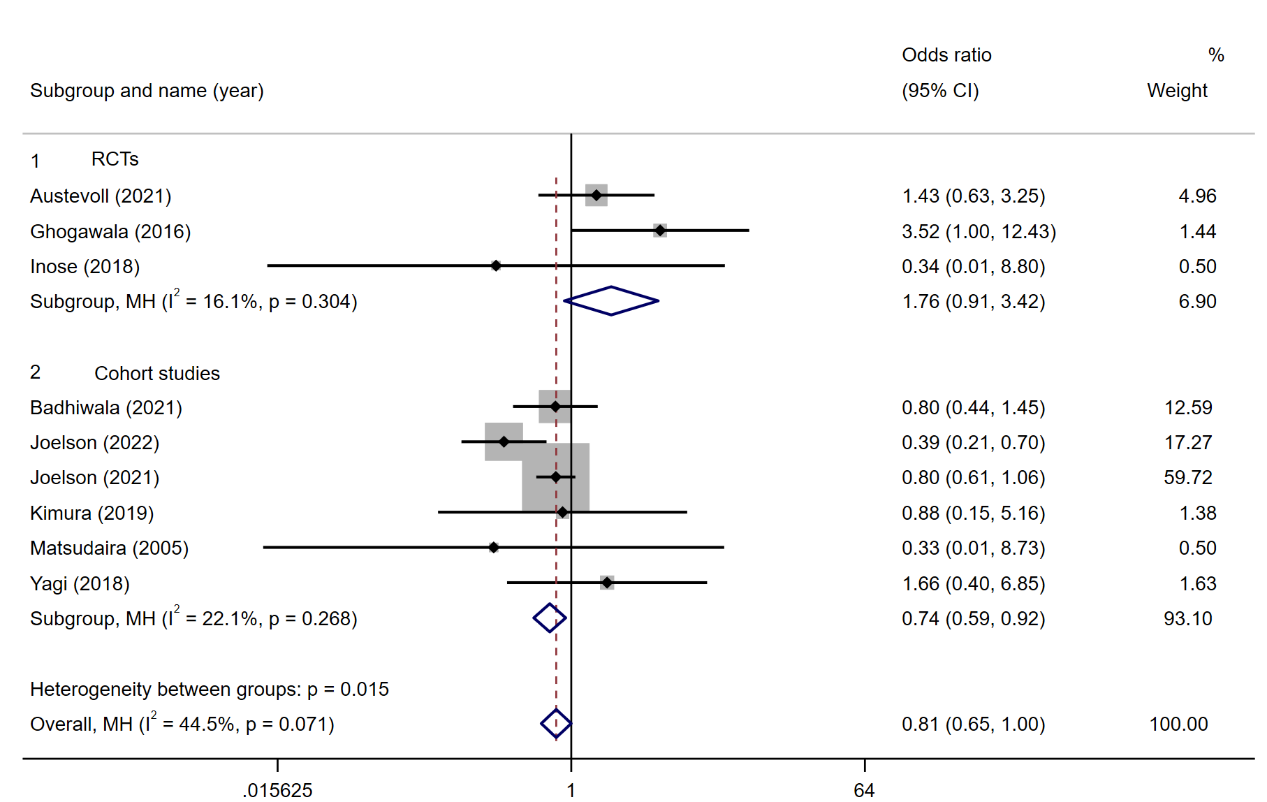


Fig.S3 Subgroup forest plot of reoperation based on article type(cohort study or RCT)


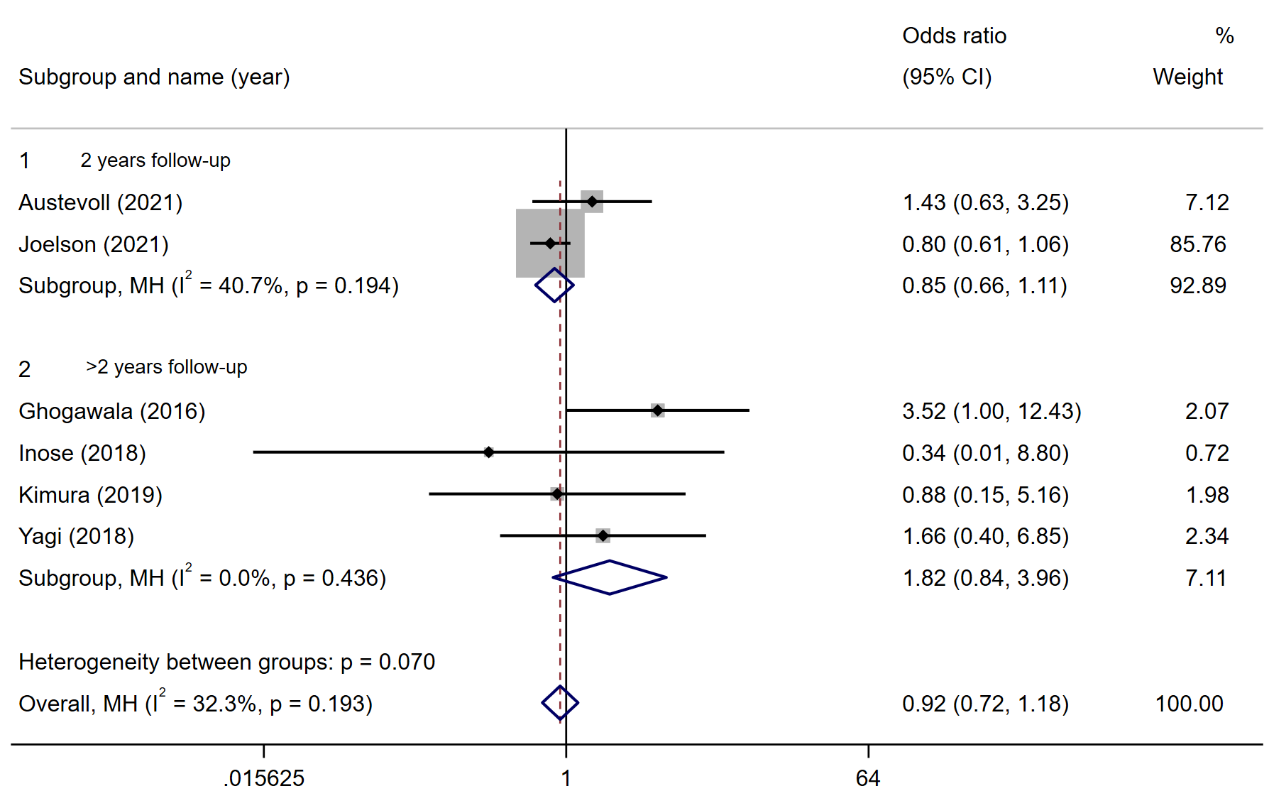


Fig.S4 Subgroup forest plot of reoperation based on follow-up time (2 years or more)


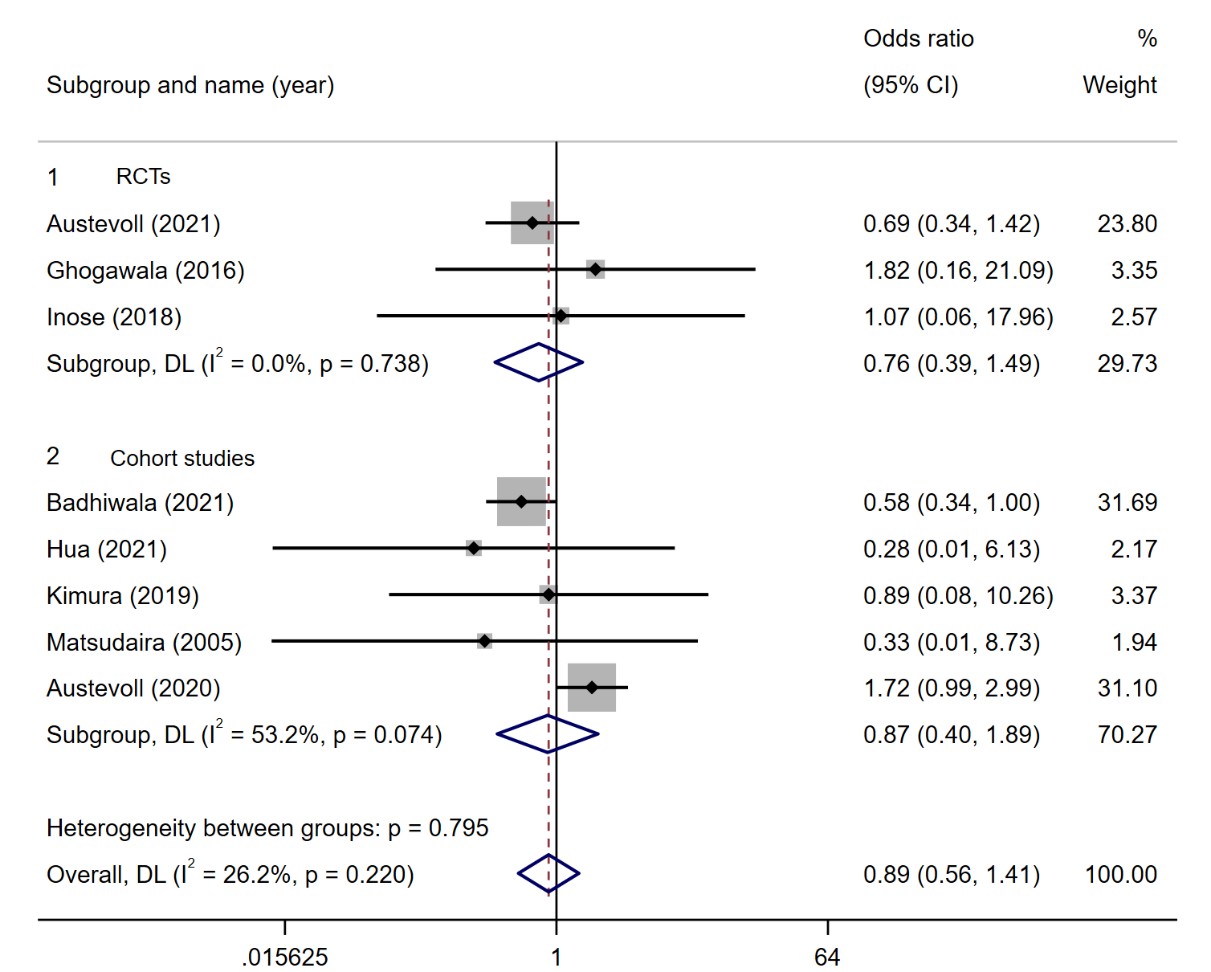


Fig.S5 Subgroup forest plot of postoperative complications based on article type(cohort study or RCT)

| **Table.S1 \| Scores of the Newcastle-Ottawa Quality Assessment Scale for 9 cohort studies** | | | | |
| --- | --- | --- | --- | --- |
| Study(year) | Selection | Comparability | Outcome | Total |
| Badhiwala(2021) | 4 | 1 | 3 | 8 |
| Hua(2021) | 4 | 2 | 3 | 7 |
| Joelson(2022) | 4 | 0 | 3 | 7 |
| Joelson(2021) | 4 | 0 | 3 | 7 |
| Kimura(2019) | 4 | 2 | 2 | 8 |
| Matsudaira(2005) | 3 | 1 | 3 | 7 |
| Sigmundsson(2015) | 4 | 2 | 3 | 9 |
| Austevoll(2020) | 4 | 3 | 2 | 9 |
| Yagi(2018) | 3 | 1 | 3 | 7 |

| **Table.S2 \| The results of publication bias by Begger’ and Egger’ test** | | | | | |
| --- | --- | --- | --- | --- | --- |
| Indicators | | Number of included studies | p values of pooled effect | Egger’ test for publication bias | Begg’ test for publication bias |
| Operation time | | 7 | p = 0.000 | P = 0.489 | P = 0.452 |
| Intraoperative Blood loss | | 5 | p = 0.000 | P = 0.098 | P = 0.734 |
| Reoperation | | 9 | p = 0.051 | P = 0.532 | P = 0.602 |
| Postoperative Complications | | 8 | p = 0.025 | P = 0.633 | P = 1.000 |
| ODI scores | 3 months | 4 | 0.623 | P = 0.560 | P = 0.499 |
|  | 1 year | 6 | 0.512 |  |  |
|  | 2 year | 5 | 0.539 |  |  |
| Back pain | 3 months | 3 | 0.786 | P = 0.066 | P = 0.107 |
|  | 1 year | 4 | 0.149 |  |  |
|  | 2 year | 3 | 0.050 |  |  |
| Leg pain | 3 months | 3 | 0.156 | P = 0.252 | P = 0.059 |
|  | 1 year | 4 | 0.807 |  |  |
|  | 2 year | 3 | 0.410 |  |  |
| *ODI*: Oswestry disability index | | | | | |
